# Absence of xenotropic murine leukaemia virus-related virus in UK patients with chronic fatigue syndrome

**DOI:** 10.1186/1742-4690-7-10

**Published:** 2010-02-15

**Authors:** Harriet CT Groom, Virginie C Boucherit, Kerry Makinson, Edward Randal, Sarah Baptista, Suzanne Hagan, John W Gow, Frank M Mattes, Judith Breuer, Jonathan R Kerr, Jonathan P Stoye, Kate N Bishop

**Affiliations:** 1 Division of Virology, MRC National Institute for Medical Research, The Ridgeway, Mill Hill, London NW7 1AA, UK; 2 CFS Group, Division of Cellular & Molecular Medicine, St George's University of London, Cranmer Terrace, London SW17 0RE, UK; 3 The Centre for Forensic Investigation, Dept of Biological and Biomedical Sciences, Glasgow Caledonian University, Glasgow G4 0BA, UK; 4 Department of Virology, Barts and The London NHS Trust, 18 Newark St, Whitechapel, London E1 2ES, UK; 5 Division of Infection and Immunity, University College London, Windeyer Building, 46 Cleveland St, London W1T 4JF, UK

## Abstract

**Background:**

Detection of a retrovirus, xenotropic murine leukaemia virus-related virus (XMRV), has recently been reported in 67% of patients with chronic fatigue syndrome. We have studied a total of 170 samples from chronic fatigue syndrome patients from two UK cohorts and 395 controls for evidence of XMRV infection by looking either for the presence of viral nucleic acids using quantitative PCR (limit of detection <16 viral copies) or for the presence of serological responses using a virus neutralisation assay.

**Results:**

We have not identified XMRV DNA in any samples by PCR (0/299). Some serum samples showed XMRV neutralising activity (26/565) but only one of these positive sera came from a CFS patient. Most of the positive sera were also able to neutralise MLV particles pseudotyped with envelope proteins from other viruses, including vesicular stomatitis virus, indicating significant cross-reactivity in serological responses. Four positive samples were specific for XMRV.

**Conclusions:**

No association between XMRV infection and CFS was observed in the samples tested, either by PCR or serological methodologies. The non-specific neutralisation observed in multiple serum samples suggests that it is unlikely that these responses were elicited by XMRV and highlights the danger of over-estimating XMRV frequency based on serological assays. In spite of this, we believe that the detection of neutralising activity that did not inhibit VSV-G pseudotyped MLV in at least four human serum samples indicates that XMRV infection may occur in the general population, although with currently uncertain outcomes.

## Background

In 2006, pursuing a link between prostate cancer and an inherited mutation in the RNASEL gene, Urisman and colleagues identified a novel gammaretrovirus [1]. Using PCR methodology, this virus was shown to be present in 9/86 (10%) prostate tumours examined. It showed close sequence similarity to xenotropic murine endogenous retrovirus elements and was thus named **x**enotropic **m**urine leukaemia virus **r**elated **v**irus (XMRV). A subsequent study demonstrated receptor usage typical of murine xenotropic virus [2]. Phylogenetic analyses place XMRV firmly within the murine endogenous retroviruses [3] even though no identical element has so far been identified within the mouse genome [4]. More recently, additional groups of samples from patients with prostate cancer have been examined for the presence of XMRV with both positive [5] and negative [6,7] results.

Very recently, a paper reporting the PCR detection of XMRV in PBMC from 68/101 patients with chronic fatigue syndrome (CFS) has been published [8]. Replicating virus could be isolated from stimulated PBMC with sequences almost, but not quite identical to the viruses isolated from prostate cancer patients. Providing apparently compelling evidence against the possibility of laboratory contamination, a number of the patients were shown to have mounted an immune response against XMRV. Interestingly, around 4% of control patients appeared to harbour the virus [8].

Replication of these results and the possible identification of roles for XMRV in the aetiology of prostate cancer and/or CFS would be of great medical significance. Detection of XMRV might provide potentially useful diagnostic tools and might also suggest therapeutic avenues for treatment. Further, widespread distribution of a potentially pathogenic virus would have important implications concerning its role as a co-factor in other conditions and in the safety of the blood supply. We therefore set out to investigate the distribution of XMRV in UK CFS patients, using PCR to search for the presence of XMRV DNA and neutralisation assays to detect an anti-XMRV immune response. In this study we did not find any association between XMRV infection and CFS.

## Methods

### Sample collection

Samples from the following three centres were tested; St George's University of London (SGUL), Barts and the London Hospital Trust (BLT) and Glasgow Caledonian University (GC).

The SGUL cohort comprised 142 adult CFS patients and 157 healthy blood donors. Both groups were aged between 18 and 65, and the male to female ratios were 45:97 (CFS) and 43:114 (blood donors). At the time of sampling, 2003-2008, blood was collected into three tubes (an EDTA blood tube for DNA preparation; a Paxgene tube for RNA preparation and a plain tube for serum preparation from clotted blood). CFS patients were recruited from clinics in Bristol, Dorset, London, Birmingham, Norfolk and Epsom, and all patients fulfilled diagnostic criteria of Fukuda *et al*. [9]. Blood samples were taken between 1.5 and 4 years following diagnosis. Healthy normal blood donors were enrolled from the National Blood Service (NBS), in Dorset, UK. All subjects provided informed consent, and these studies were approved by Wandsworth Research Ethics Committee, St George's Hospital, Cranmer Terrace, London SW17 0RE.

The BLT cohort comprised 226 anonymised serum samples taken in 2008-2009 (57 from the antenatal clinic; 58 with haematological disorders; 55 liver patients and 56 from the renal clinic). Clotted blood was separated by centrifugation, and the serum supernatant was removed, stored at -20°C and defrosted once. Ethical approval for the use of these samples for assay development was issued by UCLH NHS trust and adopted by chairman's action at BLT.

The GC cohort comprised 28 CFS patients (20 sera and 8 plasma samples) and 12 controls (8 sera and 4 plasma samples) from the West of Scotland catchment area. CFS patients were aged between 28 and 79, with a male to female ratio of 16:12. Samples were collected between 1995 and 2003. Controls were aged between 23 and 63, with a male to female ratio of 7:5. Samples were collected between 2002 and 2004. Some controls were relatives of the patients, and some were hospital staff volunteers. All patients met the Fukuda criteria (9). Ethical permission for blood samples to be analysed for the presence of viruses was granted by Southern General Hospital NHS Trust Local Ethics Committee.

### PCR

Genomic (g)DNA was prepared from PBMC from SGUL patients and controls using the QIAamp DNA mini kit (Qiagen) and amplified using the RepliG Ultrafast Mini Kit (Qiagen), which provides highly uniform amplification of all sequences, with negligible sequence bias. The concentrations after amplification ranged from 108 - 586 ng/μl. Initially, 48 CFS patient gDNA samples were screened by single-round PCR for *gag *and *env *genes, as well as GAPDH, as outlined by Lombardi *et al*. [8] (Table 1). This PCR was performed in a 50 μl reaction volume consisting of 25 μl amplitaq gold PCR mastermix and a final DNA concentration of 2-5 ng/μl. Cycling was modified as appropriate to our mastermix; 95°C for 5 min, (95°C for 30 sec, 57°C for 30 sec, and 72°C for 60 sec) for 45 cycles, hold at 72°C for 7 min, store at 4°C. Products were visualized on 3% agarose gels by ethidium bromide staining. As we did not amplify any products using this PCR, we developed two more sensitive real-time qPCR assays which targeted 2 regions of the *env *gene, beginning at nt 6173 and 6682, respectively (Table 1). These were used to screen samples of gDNA (prepared from PBMC) or cDNA (prepared from total RNA extracted using the Paxgene system from Preanalytix, UK) from CFS and normal blood donors. In total, 136 CFS gDNA and 140 CFS cDNA samples and 95 control gDNA and 141 control cDNA samples were analysed, such that all 142 CFS patients and 157 blood donors were screened for XMRV using these assays in either genomic DNA, cDNA or both. GAPDH was also amplified as a control using a commercial primer and probe set (Hs_99999905_m1 from Applied Biosystems). Real-time qPCR reactions were performed in 10 μl total volume, consisting of 5 μl PCR mastermix, 0.5 μl (20×) Taqman primers/probe mix, 4.5 μl sample (for gDNA, 1 μl gDNA (100-590 ng) and 3.5 μl DEPC-treated water (Ambion); for cDNA, 4.5 μl cDNA). Cycling times and temperatures were as follows. Initial denaturation occurred for 10 min at 95°C, followed by 40 cycles of denaturation at 95°C for 15 sec and combined primer annealing/extension at 60°C for 1 min. Data were displayed using SDS 1.3.1 software (ABI).

**Table 1:** Primer sequences used in XMRV-specific PCRs

Primer	Sequence	Reference
419F gag	ATCAGTTAACCTACCCGAGTCGGAC	Lombardi et al, 2009
1154R gag	GCCGCCTCTTCTTCATTGTTCT	Lombardi et al, 2009
5922F env	GCTAATGCTACCTCCCTCCTGG	Lombardi et al, 2009
6273R env	GGAGCCCACTGAGGAATCAAAACAGG	Lombardi et al, 2009
		
hGAPDH-66F	GAAGGTGAAGGTCGGAGTC	Lombardi et al, 2009
hGAPDH-291R	GAAGATGGTGATGGGATTTC	Lombardi et al, 2009
		

**Real-time PCR**		
6173 env F	GGCATACTGGAAGCCATCATCATC	
6173 env R	CCTGACCCTTAGGAGTGTTTCC	
6173 env probe	ATGGGACCTAATTTCC	
		
6682 env F	GTGCTGGCTGTGTCTAGTATCG	
6682 env R	GCAGAGGTATGGTTGGAGTAAGTAC	
6682 env probe	ACGGCCACCCCTTCGT	

### Plasmids

VP62 XMRV clone was a gift of Robert Silverman [2]. HG1 is a replication-incompetent XMRV clone constructed by site-directed mutagenesis of VP62 (the packaging signal was removed by deleting nucleotides 293-388, as numbered in GenBank EF185282; and nucleotides 7720-8108 were replaced by a BsrG1 site to remove the U3 region). Moloney-MLV Gag-Pol was expressed from KB4, a vector synthesized by cloning the *gag-pol *region from pMD-MLV GagPol [10] into pcDNA3.1. Viral genomic RNA was expressed from an MLV-based retroviral vector encoding β-galactosidase (LTR-LacZ [10]), and envelope proteins were encoded by constructs for either NZB xenotropic envelope, MLV(X) (a gift of Massimo Pizzato), Moloney-MLV env (MOSAF, a gift of Yasu Takeuchi), Friend-MLV env [10], or the G-protein from vesicular stomatitis virus (VSV-G) [11].

### Virus production

Replication defective XMRV virus was prepared for neutralisation assays by co-transfecting 293T cells with HG1 and LTR-LacZ. Pseudotyped MLV was prepared by co-transfecting 293T cells with KB4, LTR-LacZ and an envelope-encoding plasmid (either MLV(X), MOSAF, Friend or VSV-G). After ~18 hours, cells were washed, and fresh media was added for a further ~24 hours, before viral supernatants were harvested, filtered, and the viral titre was measured by ELISA for RT activity (Cavidi tech). Viral stocks were titrated on D17 cells, an established, easily infectable dog cell line, or NIH-3T3 cells for Friend- and Moloney- pseudotyped MLV. After 48 hours, the cells were assayed for β-galactosidase activity using the Galacto-Star system (Applied Biosystems). The amount of virus to be used in the neutralisation assays was determined as the volume of supernatant added to 3.5 × 10^3 ^cells that resulted in ~4 × 10^4 ^counts per second of chemiluminescence.

### Neutralisation assays

Neutralisation assays were performed as reported in [12]. Monoclonal antibodies to MLV Env proteins (shown in Table 2) were gifts from Leonard Evans and have been previously described [13,14]. They were provided and used as untreated hybridoma cell supernatants that were serially diluted two-fold before adding to virus to assess neutralisation activity as for serum, detailed below. Serum samples were heat inactivated at 56°C for 30 min. 5 μl serum were then added to 95 μl media in a 96-well tissue culture plate, and samples were serially diluted two-fold, leaving 50 μl at each dilution. 50 μl virus-containing supernatant were then added to each well, and the plate was incubated at 37°C for 1 hour. Following incubation, 100 μl containing 3.5 × 10^3 ^D17 cells (or NIH-3T3 cells for Friend or Moloney-MLV neutralisation) were added to each well, and the plate was incubated at 37°C. After 48 hours the cells were lysed, and β-galactosidase activity was measured. Infectivity corresponded to counts per second of chemiluminescence.

**Table 2:** Neutralisation properties of different monoclonal antibodies against XMRV and MLV pseudotyped with three different envelopes.

			Neutralisation of			
**Hybridoma**^**1**^	Raised in	Isotype	XMRV	MLV(X)	Friend	Moloney
83A25'	Rat	IgG2A	Y (88)	Y (89)	ND	ND
24-7	Mouse	IgMK	N	N	ND	ND
48	Mouse	IgG2A	N	N	Y (95)	Y (83)
538	Mouse	IgM	N	N	N	Y (63)
603	Mouse	IgM	N	Y (96)	N	ND
609	Mouse	IgM	Y (71)	N	ND	ND
610	Mouse	IgM	N	Y (64)	ND	ND
613	Mouse	IgM	N	Y (91)	ND	ND
615	Mouse	IgM	N	N	ND	ND

Y indicates neutralisation; N indicates no neutralisation; ND is not determined

The number in brackets refers to the percentage neutralisation at the least diluted antibody concentration.

^1 ^See references [13] and [14] for description of hybridoma cell lines

## Results

### PCR screening

**Figure 1:**
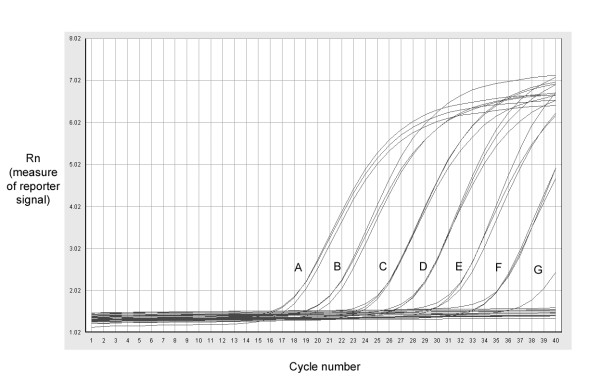
**Sensitivity of PCR screening for XMRV in PBMC DNA**. VP62 plasmid was serially diluted 1:10 into PBMC DNA from a healthy donor and tested by Taqman PCR with env 6173 primers and probe. The final amount of VP62 DNA in the reaction was A, 2.3 × 10^-2 ^ng, B, 2.3 × 10^-3 ^ng, C, 2.3 × 10^-4 ^ng, D, 2.3 × 10^-5 ^ng, E, 2.3 × 10^-6 ^ng, F, 2.3 × 10^-7 ^ng or G, 2.3 × 10^-8 ^ng. The limit of sensitivity was 2.3 × 10^-7 ^ng (shown by trace F) which equates to 16 molecules of VP62 XMRV clone.

Lombardi *et al*. have recently detected XMRV DNA in 67% of CFS patients by PCR [8]. To confirm an association of XMRV with this disease, we performed PCR for *gag*, *env *and GAPDH on 48 (of 142) CFS patient gDNA samples from SGUL using the previously published single-round PCR methodology (Table 1 and [8]. However, although all samples were positive for GAPDH, we found no evidence of XMRV DNA in any of the samples (data not shown). In case we were missing low levels of viral DNA, we devised a more sensitive qPCR-based approach. To test the sensitivity of this method, triplicate, serial 1:10 dilutions of VP62 plasmid encoding the full length XMRV genome were added to PBMC DNA from a healthy donor and tested by Taqman PCR with either env 6173 or env 6682 primers (Table 1). All replicates calculated to contain 16 copies of XMRV routinely yielded a product within 37 cycles whereas only one of three replicates of the next dilution scored positive (Figure 1). We concluded that our assay was capable of reliably detecting as little as 16 copies of proviral DNA and was therefore likely to be as sensitive, if not more so, than the assays previously used [8]. We then tested the entire SGUL panel of 142 CFS samples and 157 of the control samples (either gDNA, cDNA or both) with both env 6173 and env 6682 primers. Although positive for GAPDH, all samples were negative for XMRV. To exclude the possibility of specific sample-mediated PCR inhibition, we spiked 3 normal control cDNAs, which had previously tested negative for XMRV nucleic acid, with XMRV VP62 DNA, to a final concentration of 2.3 × 10^-6 ^ng/μl and repeated the qPCR using both env 6173 and env 6682 primer sets. We successfully amplified the VP62 in these reactions, proving that the PCR should have amplified XMRV from the patient samples if it was present.

### Neutralisation assays

**Figure 2:**
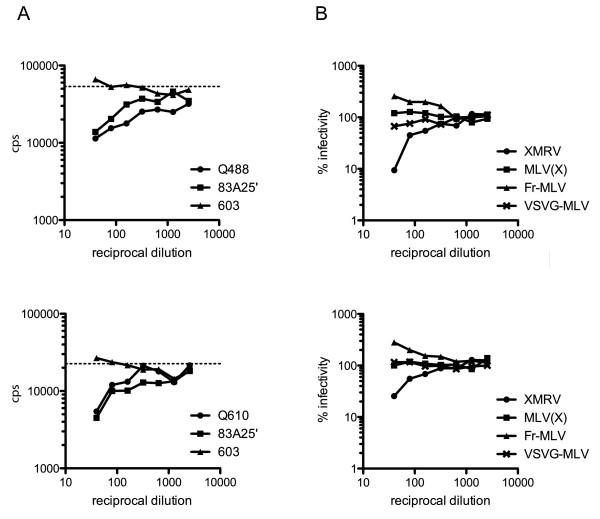
**Examples of BLT positive serum neutralisation activity**. A, Infectivity of XMRV (measured as counts per second of chemiluminescence produced from β-galactosidase activity) after incubation with patient serum or hybridoma cell supernatant. Infectivity is plotted against the reciprocal dilution of the BLT serum (black circles, top panel, sample Q488, bottom panel, sample Q610; triangles, negative control, monoclonal 603; squares, positive control, monoclonal 83A25'). The dashed line indicates viral infectivity in the absence of sera. B, Infectivity data for viruses with four different envelopes (circles, XMRV; squares, MLV(X); triangles, Friend-MLV; crosses, VSV-G) after incubation with patient serum. Data were normalised by setting the infectivity for each virus in the absence of patient serum at 100%, and plotted against the reciprocal of serum dilution for two positive sera, top panel sample Q488 and bottom panel sample Q610.

**Figure 3:**
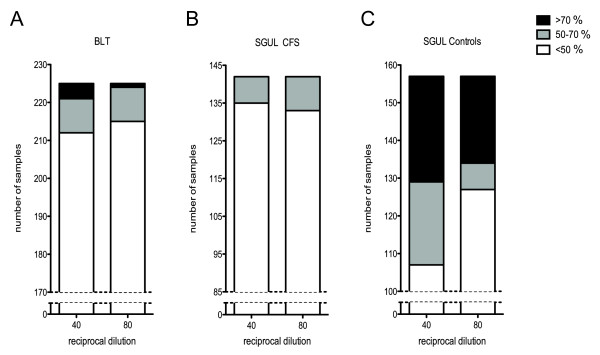
**Distribution of neutralisation activity in three samples sets**. Numbers of patients showing different degrees (>70% , 50-70% and <50%) of neutralisation of XMRV infectivity are shown for the 1/40 and 1/80 serum dilutions. A, Total BLT cohort (n = 226); B, SGUL CFS cohort (n = 142); C, SGUL control blood donor cohort (n = 157).

In light of the negative data obtained using PCR assays, we set out to search for evidence of XMRV infection using a second method. Viral infection can elicit a neutralising antibody response [12]. Demonstration of such a neutralising activity can be taken as evidence for a viral infection, perhaps in cell types that were not sampled in blood. Defining neutralisation is difficult in the absence of known positive and negative sera. However, a number of neutralising monoclonal antibodies directed against the Env protein of murine retroviruses have been described [13,14]. We therefore obtained several of these (gifts of Leonard Evans) and tested them for neutralisation of XMRV and NZB xenotropic MLV(X) as well as ecotropic Friend and Moloney MLV (Table 2) by assaying for a reduction in virus infectivity following incubation of virus-containing supernatant with the monoclonal antibody. As anticipated, some monoclonal antibodies were able to neutralise XMRV (83A25' and 609) whilst others had no effect on XMRV infectivity. Interestingly, we identified three monoclonal antibodies that neutralised MLV(X) but not XMRV (603, 610 and 613) and one that neutralised XMRV but not MLV(X) (609). These reagents may therefore be useful tools with which to distinguish XMRV from other xenotropic MLVs in future investigations. From these experiments we defined two negative (603 and 613) and one positive (83A25') antibody controls for further experiments. To validate the neutralisation assay and examine the possible range of responses to "normal serum", we tested neutralisation using a panel of 226 serum samples from BLT. Previous investigations have detected XMRV DNA in ~1-6% of control samples [5,6,8]. Of our panel only a handful showed possible neutralisation activity, giving curves similar to that shown in Figure 2A, with reductions in viral infectivity similar or greater than that seen with the positive control, monoclonal 83A25'. Over 90% of the samples tested had less than a 2-fold effect on infectivity (Figure 3A). From these data, we have defined a positive as a sample that reduces viral infectivity by at least 70% at a dilution of 1/40 and gives a reduction of 50% at a 1/80 dilution. According to this definition, the BLT sample set contains 3 neutralising sera, identifying 1.3% of samples as positive.

To confirm that the neutralisation activity demonstrated was specific for XMRV, we tested a subset of sera for neutralisation of XMRV alongside MLV particles pseudotyped with different envelope proteins from MLV(X), Friend-MLV or VSV. As shown in Figure 2B, of these four virus preparations, only XMRV infectivity was inhibited by any of the sera tested. Even the infectivity of particles expressing the closely related MLV(X) envelope that is 94% identical to XMRV was unaffected by sera that inhibited XMRV (Figure 2B, squares). Thus, it seems that the neutralising activity is specific for XMRV.

**Figure 4:**
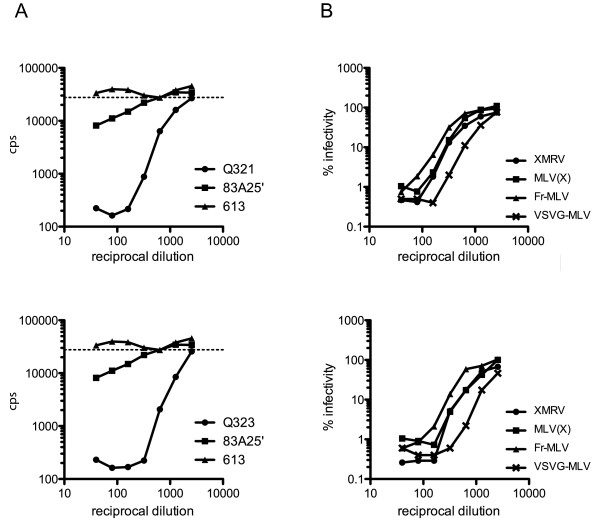
**Examples of SGUL positive serum neutralisation activity**. A, Infectivity of XMRV (measured as counts per second of chemiluminescence produced from β-galactosidase activity) after incubation with patient serum or hybridoma cell supernatant. Infectivity is plotted against the reciprocal dilution of the SGUL serum (black circles, top panel, sample Q321, bottom panel, sample Q323; triangles, negative control, monoclonal 613; squares, positive control, monoclonal 83A25'). The dashed line indicates viral infectivity in the absence of sera. B, Infectivity data for viruses with four different envelopes (circles, XMRV; squares, MLV(X); triangles, Friend-MLV; crosses, VSV-G) after incubation with patient serum. Data were normalised by setting the infectivity for each virus in the absence of patient serum at 100%, and plotted against the reciprocal of serum dilution for two positive sera, top panel sample Q321 and bottom panel sample Q323.

We therefore felt this assay was sensitive and specific enough to examine the neutralising ability of the SGUL cohort of blinded patient serum samples. After unblinding the samples, it emerged that of the 142 CFS patient sera tested none was positive as defined by the criteria above (Figure 3B). These results suggested that there was no link between XMRV and CFS. By contrast, the control group of 157 blood donors contained 22 positives, a frequency of 14%, considerably higher than that seen in the BLT group (Figure 3C). It was also noticeable that the neutralising activity of all but one of the SGUL positive samples was much stronger than the BLT positive samples (compare Figure 2A with Figure 4A). In fact, most of the SGUL positive sera reduced XMRV infectivity by 100 fold at both 1/40 and 1/80 dilutions. Intriguingly, many of these serum samples were collected from a single blood donation session. Some samples from this session, however, were negative. Surprisingly, PCR analyses of DNA samples corresponding to the positive sera from the SGUL controls were uniformly negative. We therefore investigated the specificity of this response by testing 21 of the positive sera for neutralisation of MLV pseudotyped with the envelope proteins from MLV(X), Friend-MLV or VSV. In every case, the serum was able to neutralise additional viruses to XMRV, including particles pseudotyped with the non-retroviral envelope from VSV (Figure 4B and Table 3). This implied that the strong positive neutralising activity demonstrated by the SGUL blood donor controls was not specific to XMRV, and in all likeliness was not elicited by this virus.

**Table 3:** Neutralisation properties of different human sera against XMRV and MLV pseudotyped with three different envelopes.

	Neutralisation of				
Sample ID	XMRV	MLV(X)	Friend	VSV	XMRV detected by PCR
Barts and the London					
Q488	+	-	-	-	ND
Q610	+	-	-	-	ND
Q663	+	ND	ND	ND	ND
St George's University of London					
Q302	++	++	++	++	no
Q304	++	++	++	++	no
Q305	++	++	++	++	no
Q306	++	++	++	++	no
Q307	++	+	+	-	no
Q308	++	++	++	++	no
Q309	++	++	++	++	no
Q310	++	++	++	++	no
Q311	++	+	+	+	no
Q312	++	++	++	++	no
Q313	++	++	++	++	no
Q314	++	ND	ND	++	no
Q315	++	++	++	++	no
Q316	++	++	++	++	no
Q317	++	++	++	++	no
Q319	++	ND	ND	++	no
Q320	++	++	++	++	no
Q321	++	++	++	++	no
Q323	++	++	++	++	no
Q324	++	++	++	++	no
Q326	++	ND	ND	ND	no
Q372	+	-	-	+	no
Glasgow Calendonian University					
Q125	+	++	++	-	ND

+ indicates neutralising activity; ++ indicates strong neutralising activity; - indicates no neutralising activity; ND is no determined.

To test whether the SGUL cohort of CFS patients was unique, we also tested 40 samples (including some plasma samples as well as sera) from a separate CFS cohort in our neutralisation assay. This GC cohort revealed a solitary positive out of 28 CFS samples (3.6%), and no positives out of 12 control samples. The positive CFS patient serum was also able to neutralise MLV pseudotyped with either MLV(X) or Friend envelopes, although interestingly, it was not able to neutralise VSV-G pseudotyped MLV (Table 3). Neutralisation data from the different cohorts are summarized in Table 4. Thus, in summary, we found no association of XMRV with either CFS cohort.

**Table 4:** Summary of number of positive sera with XMRV neutralisation properties

Sample cohort	Positive	Total number
Barts and the London		
Control	3	226
St Georges University of London		
CFS	0	142
Control	22	157
Glasgow Caledonian University		
CFS	1	28
Control	0	12

## Discussion

We set out with the intention of confirming the results of Lombardi *et al*. [8] concerning the association of XMRV with CFS. In total, we tested 142 CFS samples for both the presence of XMRV DNA in PBMCs by PCR and for the presence of neutralising antibodies against XMRV in our viral neutralisation assay, and a further 28 CFS samples for neutralising antibodies only. However, in contrast to Lombardi *et al*., we found no evidence of XMRV DNA in any patient samples tested, and only a single neutralisation-positive patient serum. Our findings therefore appear inconsistent with the previous report that isolated XMRV from PBMCs of CFS patients. We are confident that, although we are unable to replicate the PCR detection of XMRV in PBMC DNA from CFS patients, our PCR assay is more sensitive than the published single round PCR method and should have possessed the necessary sensitivity to detect XMRV if it was indeed present (Figure 1). Furthermore, we were able to detect neutralising activity in one patient and in several control serum samples (Table 4 and Figure 3), implying that our neutralisation assay also has the required sensitivity. The lack of neutralising activity in CFS samples compared to controls could reflect an inability to mount an immune response in these patients. However, in that case, the virus would be expected to replicate to higher levels in CFS patients making it easier to detect by PCR. As we could not detect any evidence of XMRV infection by our PCR assays, we think this is an unlikely explanation. Thus, in our cohorts, we found no association of XMRV with CFS. This is in stark contrast to the result of Lombardi *et al*. [8]. However, it is thought likely that the term CFS defines multiple diseases [15-17], and it remains formally possible that a fraction of these are associated with XMRV. During the submission of this manuscript another report was published by Erlwein *et al*. that also failed to detect XMRV in CFS patients by PCR [18]. The publication of these results has promoted much discussion and controversy amongst CFS researchers and patients alike, and has highlighted the need for additional investigations in this area. Following the findings reported here, it would seem a prudent next step for subsequent studies to compare samples and protocols between different laboratories around the world.

There have also been conflicting reports describing the association of XMRV with prostate cancer. Two studies from the USA [1,5] have found an increased prevalence of the virus in prostate cancer patients, although they differed as to whether this was dependent on the RNASEL genotype of the patient. Conversely, two German studies failed to establish a link between the virus and disease [6,7]. Nevertheless, XMRV has been detected in the control groups in multiple investigations [5,6,8], with the incidence varying between 1 and 6%. In our serological studies we have also identified neutralising activity against XMRV in around 4% of all the samples examined. Remarkably many (but not all) of the seropositive samples were identified in a relatively small group of blood donors within the SGUL cohort, possibly suggesting a local outbreak of infection. There is no evidence that this group are related or that they have a particularly high risk of acquiring a retroviral infection. Therefore, an outbreak of this kind seems unlikely. Moreover, all but one of the positive samples from the SGUL set we tested were also able to neutralise MLV pseudotyped with the envelope protein from VSV (Table 3). The one serum that failed to neutralise VSV-G pseudotyped MLV was, however, able to neutralise MLV particles pseudotyped with other retroviral envelopes. We therefore consider these positives from healthy blood donors to be non-specific cross reacting responses. The remaining four positive samples from the BLT and GC cohorts had much weaker neutralisation activities and did not neutralise VSV-G pseudotyped MLV, although, again, the positive serum from GC did neutralise particles expressing other retroviral envelopes (Table 3). Although we cannot rule out the possibility that the activity of these samples against XMRV is also non-specific, one possible explanation for these serological findings remains that XMRV infection has occurred in around one percent of the population. This figure is consistent with the general prevalence in control samples previously reported. Given the common oncogenic properties of gammaretroviruses [19] and the reported link between XMRV and prostate cancer [1,5], such an observation might be of considerable significance, particularly for the blood transfusion services. It should, however, be noted that we have so far been unable to reliably detect bacterially expressed XMRV Gag proteins by using these sera in immunoblotting experiments. It is therefore conceivable that these neutralising activities were not elicited by XMRV. Further investigations are required to determine the nature of these antiviral activities.

## Conclusions

In summary, we have studied 299 DNA samples and 565 serum samples for evidence of XMRV infection. We have not identified XMRV DNA in any samples by PCR, however, some serum samples were able to neutralise XMRV infectivity in our assay. Only one of these positive sera came from a CFS patient, implying that there is no association between XMRV infection and CFS. Furthermore, most of the positive sera were also able to neutralise MLV particles pseudotyped with other envelope proteins, indicating there may be cross reactivity with other retroviruses and even other enveloped viruses. It therefore seems unlikely that these responses were elicited by XMRV. However, the detection of neutralising activity that did not neutralise VSV-G pseudotyped MLV in at least four human sera may indicate that XMRV infection does occur at in the general population, although the outcome of such infections is currently uncertain.

## Competing interests

The authors declare that they have no competing interests.

## Authors' contributions

JK, JS and KB conceived and designed the investigation. HG and VB carried out the viral neutralisation assays and analysed the data. KM, ER, SB and JK performed the PCR analyses. SH, JG, FM, JB and JK provided patient samples. JS and KB analysed the data and drafted the manuscript. All authors read and approved the final manuscript.
